# The toxin from the PemIK-Sa1 toxin-antitoxin system decreases *Staphylococcus aureus* resistance to antibiotics

**DOI:** 10.3389/fmicb.2026.1863659

**Published:** 2026-07-31

**Authors:** Emilia Bonar, Kinga Chlebicka, Michał Bukowski, Weronika Jodłowska, Maja Kosecka-Strojek, Anna Rabięcny, Piotr Suder, Małgorzata Hopciaś, Zuzanna Głowacka-Grzyb, Viktoria Akkerboom, Andreas Voss, Artur J. Sabat, Benedykt Władyka

**Affiliations:** 1 Department of Analytical Biochemistry, Faculty of Biochemistry, Biophysics and Biotechnology, Jagiellonian University, Krakow, Poland; 2 Department of Medical Microbiology and Infection Prevention, University Medical Center Groningen, University of Groningen, Groningen, Netherlands; 3 Department of Microbiology, Faculty of Biochemistry, Biophysics and Biotechnology, Jagiellonian University, Krakow, Poland; 4 Department of Analytical Chemistry and Biochemistry, Faculty of Materials Science and Ceramics, AGH University of Krakow, Krakow, Poland; 5 Doctoral School of Exact and Natural Sciences, Jagiellonian University, Kraków, Poland

**Keywords:** antibiotic resistance, proteomics, RNase, *Staphylococcus aureus*, toxin-antitoxin system, transcriptomics

## Abstract

Toxin-antitoxin (TA) systems are bacteria-specific components involved in maintaining mobile genetic elements that may carry antibiotic resistance determinants (ARDs), among other functions. PemIK-Sa1 is a plasmid-encoded TA system found in *Staphylococcus aureus*, a dangerous pathogen affecting humans and animals. The PemK-Sa1 toxin is a sequence-specific ribonuclease inhibited by its cognate antitoxin, PemI-Sa1. Using recombinant multidrug-resistant strains, we demonstrated that expression of the toxin reduces resistance to antibiotics from various classes, including β-lactams, fluoroquinolones, aminoglycosides, and chloramphenicol. Transcriptomics revealed that, in addition to downregulation of genes encoding ARDs, nearly one-third of the staphylococcal transcriptome is altered. Furthermore, proteomic analysis revealed that toxin expression results in the downregulation of several proteins involved in nucleotide biosynthesis, carbohydrate metabolism, and energy production. This indicates parallel mechanisms in which the toxin not only degrades ARD transcripts, thereby directly impacting their function, but also alters the expression of genes encoding proteins crucial to essential cell processes. The ability to reduce resistance to a broad range of antibiotics makes the PemK-Sa1 toxin a promising tool for combating multidrug-resistant bacteria through the artificial activation of the PemIK-Sa1 TA system.

## Introduction

*Staphylococcus aureus* is an opportunistic pathogen of humans and farm and livestock animals (Haag et al., 2019). In humans, the bacteria often asymptomatically colonize mainly the skin and mucous membranes of the upper respiratory tract. However, they also cause infections of diverse sites, clinical presentations, and severity, ranging from minor skin lesions and postoperative wound infections to gastrointestinal and respiratory disease, and ultimately to life-threatening conditions such as toxic shock syndrome, endocarditis, meningitis, and sepsis (Cheung et al., 2021; Lakhundi and Zhang, 2018). The pathogenicity of *S. aureus* results from its ability to synthesize a wide range of virulence factors, including surface proteins, extracellular enzymes, and toxins (Bien et al., 2011). Moreover, *S. aureus* can employ several strategies that make it resilient and adaptive to the host immune response, including the formation of biofilms and persister cells. As a consequence, it enhances its survival in unfavorable conditions and causes difficulties in combating the infection. However, even more important from a medical perspective is its antibiotic resistance. In 2019, *S. aureus* was associated with more than 1 million deaths globally (Ikuta et al., 2022), and the emergence of highly pathogenic and multidrug-resistant strains poses a growing threat to the healthcare system (Lee et al., 2018). Virulence factors and antibiotic resistance determinants are often located on mobile genetic elements (MGEs) such as plasmids, transposons, and pathogenicity islands. This facilitates their acquisition and spread by horizontal gene transfer (Van Hoek et al., 2011). The highly successful strain *S. aureus* USA300 has integrated the arginine catabolic mobile element (ACME) downstream of the staphylococcal chromosomal cassette (SCC) harboring the methicillin resistance gene *mecA* (SCC*mec*). Moreover, the pathogenicity island, SaPI5, encodes two enterotoxins while prophages ϕSA2usa and ϕSa3usa harbor the genes that encode the Panton-Valentine leucocidin (PVL) and staphylokinase and a chemotaxis inhibiting protein, respectively. The combination of these chromosomal elements with plasmids pUSA02 and pUSA03 providing resistance to tetracycline, erythromycin, clindamycin, streptogramin B, and mupirocin, renders this strain a highly pathogenic and multidrug-resistant superbug (Diep et al., 2006).

Toxin-antitoxin (TA) systems are small genetic elements widespread in bacteria. They were shown to be involved in the maintenance of MGEs, biofilm and persister cell formation, protection against phage infection, and virulence in case of pathogens (Jurėnas et al., 2022; Singh et al., 2021). TA systems are classified into eight types based on the nature of antitoxins and the mode of action. However, regardless of the type, the antitoxin inhibits the expression or activity of the toxin. An imbalance in TA system components leads to the release of toxins, causing cell poisoning by altering vital processes such as transcription and protein synthesis (Boss and Kędzierska, 2023; Jurėnas et al., 2022). This results in growth inhibition and metabolic dormancy, which may be advantageous in coping with stress conditions such as exposure to antibiotics, as suggested for the persister cell phenotype (Wood et al., 2013). On the other hand, the burden caused by the toxin reduces the bacterial fitness and, as a result, their competitiveness with the TA system-negative ones (Bukowski et al., 2025).

*Staphylococcus aureus* carries a few TA systems belonging to types I and II (Schuster and Bertram, 2016). Among the type II systems are highly prevalent chromosomally encoded MazEF, YefM/YoeB-Sa1 and YefM/YoeB-Sa2, and less frequent plasmid-encoded PemIK-Sa1 (Bukowski et al., 2017). YoeB-Sa1 and YoeB-Sa2 are ribosome-dependent RNases that cleave mRNA near the start codon (Bukowski et al., 2013). MazF and PemK-Sa1 toxins are ribosome-independent sequence-specific RNases toward UACAU and UAUU, respectively (Zhu et al., 2009; Bukowski et al., 2013). The tetrad sequence recognized by PemK-Sa1 is underrepresented in transcripts of genes encoding virulence factors, whereas it is overrepresented in mRNAs for transmembrane transporters. The susceptibility of transcripts to cleavage by the toxin correlates positively with the number of UAUU sequences (Bukowski et al., 2013). Moreover, ectopic expression of the toxin decreases resistance to chloramphenicol in *S. aureus* (Bukowski et al., 2025). However, the overall influence of PemK-Sa1 on the transcriptome and antibiotic resistance has not been experimentally verified. Interestingly, non-aureus staphylococci also carry chromosomally encoded homologs of the PemIK system. The PemIK-Sa6 system is encoded within SCC*mec* of the methicillin-resistant *S. pseudintermedius* A116 strain (Bukowski et al., 2017). However, the toxin is non-functional due to a lack of ribonuclease activity. This corresponds with the recent finding that type II TA systems, where the toxins are RNases, correlate negatively with antibiotic resistance determinants (Bukowski et al., 2025). The toxin PemK-Sa1Sp, encoded by all *S. pseudintermedius* strains, exhibits RNase activity *in vitro*; however, it is non-toxic to the host upon activation (Janczak et al., 2020). The rationale for this phenomenon remains unknown.

In this study, we investigated how the PemK-Sa1 toxin influences antibiotic resistance in *S. aureus*. Using transcriptomics and proteomics, we identified genes whose expression is affected by the toxin. PemK-Sa1 significantly reduces resistance to β-lactams, fluoroquinolones, aminoglycosides, and chloramphenicol. This phenotypic effect was confirmed by transcriptomic analysis, which revealed that PemK-Sa1 influences the transcription of approximately one-third of the genome. Furthermore, the expression of PemK-Sa1 leads to the downregulation of proteins involved in amino acids, nucleotides, and carbohydrate transport and metabolism. We propose that reduced antibiotic resistance in *S. aureus* expressing the PemK-Sa1 toxin is attributable to two mechanisms. First, the toxin directly affects the transcripts of antibiotic-resistance genes. Second, it affects gene transcripts encoding proteins that regulate gene expression, protein synthesis, and transmembrane transport, ultimately reducing the overall fitness of the bacteria.

## Materials and methods

### Bacterial strains and plasmids

*Staphylococcus aureus* 3956/13/K and CH24 (genome accession no. MOYJ00000000) are MRSA strains isolated from a human patient and a chicken, respectively (Bonar et al., 2016; Kosecka-Strojek et al., 2020). The complete genome of 3956/13/K was obtained in this study using the methodology described elsewhere (Sabat et al., 2022) and deposited under BioProject accession number PRJNA1218464, assembly accession GCA_054911445.1, sequence accession version numbers: CP180165.1–68.1 (chromosome, pAH5667, pBU108a, and pUSA395613, respectively). The corresponding raw Illumina and Nanopore reads are available in the NCBI Sequence Read Archive (SRA) under the same BioProject accession number. The pCN51 plasmid (cadmium-inducible promoter, erythromycin resistance) (Charpentier et al., 2004) was used to construct vectors for PemIK-Sa1 and PemK-Sa1 expression (Bukowski et al., 2013). *Staphylococcus aureus* strains were transformed by electroporation. Transformants were selected and cultivated in tryptic soy broth supplemented with erythromycin 10 μg/mL (TSBerm10). Recombinant expression was induced with CdCl_2_, where indicated.

### Material for transcriptomic and proteomic analysis

A single colony of *S. aureus* 3956/13/K carrying pCN51/Ø or pCN51/pemKSa1 was inoculated into 20 mL of TSBerm10 and cultured overnight. Then, cultures were diluted with fresh TSBerm10 to an OD600 of 0.08 and grown to an OD600 of 0.8. PemK-Sa1 expression was then induced with 2.5 μM CdCl_2_. Samples for RNA and protein isolation were collected at 45 and 90 min after induction, respectively, by centrifuging 4 mL aliquots of culture (5 min, 5,000 g, 4 °C).

### Total RNA extraction, mRNA purification, RNA-Seq, and gene expression analysis

Total RNA was extracted from 4 mL cultures using TRIzol Reagent paired with Lysing Matrix B homogenization (Precellys Evolution). RNA was then treated with DNase on-column and purified using the GeneJET RNA Purification Kit. Quantity and quality were evaluated with Qubit 4.0 and the Agilent 2100 Bioanalyzer; only samples with RIN values above 9 were included. rRNA was depleted using the RiboMinus Bacteria 2.0 Transcriptome Isolation Kit (Invitrogen). Libraries were prepared with the TruSeq Stranded mRNA Kit (Illumina) and sequenced on an Illumina MiSeq (MiSeq reagent kit v3, 2 × 75 bp). Sequencing data analysis was carried out as described in detail elsewhere (Bukowski et al., 2022). The corresponding raw reads are available in the NCBI SRA under the BioProject accession number PRJNA1218464. Read quality was assessed with fastp 1.0 (Chen, 2025). Reads were then mapped to transcript sequences derived from *S. aureus* 3956/13/K genome (Supplementary material: USA300K_transcripts.fna) using Salmon 1.10 (Patro et al., 2017). Differential gene expression was modeled using Bioconductor DESeq2 1.42 (Love et al., 2014) and apeglm 1.24 (Zhu et al., 2019) libraries. The complete pipeline was executed using Nextflow 24.10 (DI Tommaso et al., 2017) and is available at https://github.com/michalbukowski/rnaseq-pipeline-2.

### Cellular protein isolation, 2D-DIGE, and proteome analysis

Proteins from early log-phase cells were extracted using TRIzol Reagent with Lysing Matrix B homogenization. Samples were then purified, solubilized in a buffer containing urea, thiourea, and CHAPS, and quantified by the Bradford assay. Proteins were fluorescently labeled, separated by 2D DIGE, and analyzed with DeCyder 7.2. Statistical significance was assessed using Student’s *t*-test and one-way ANOVA (*p* ≤ 0.05) (Bonar et al., 2020). Differential spots (≥1.5-fold change) were excised and analyzed using a standard protocol (Lorencik et al., 2025). Briefly, spots were destained, proteins were reduced, alkylated, and digested with trypsin. NanoLC-MS/MS analysis was performed using an Ultimate 3000 system coupled to an Exploris 240 mass spectrometer (Thermo Fisher Scientific, Bremen, Germany). The setup consisted of a trap cartridge and an analytical capillary column, both packed with Acclaim PepMap C18 stationary phase. A 33-min Long gradient was applied (5%–30% acetonitrile in water, both containing 0.1% formic acid). Raw data were converted into *.mgf files and searched using Mascot Search Engine (version 2.8.0) against two databases: Swiss-Prot and S_aureus_USA300. All proteomic data, including *.raw and *.mgf files and search results, are available in ProteomeXchange/PRIDE repository under accession number: PXD077076. A detailed description of the proteomic analysis methodology is provided in the Supplementary material.

### Determination of MICs

MICs were determined following EUCAST and CLSI standards. Bacteria were cultivated in Mueller-Hinton Broth (MHB) supplemented with erythromycin (5 μg/mL) (180 rpm, 17 h, 37 °C), then diluted into fresh medium to an OD600 of 0.04. Fifty microliters of bacterial suspension were mixed with 50 μL of the tested antibiotic (penicillin, oxacillin, chloramphenicol, ciprofloxacin, kanamycin, or fusidic acid) at various discrete concentrations in MHB containing 0.5 μM CdCl2 in 96-well plates. After a 20-h incubation at 37 °C, MIC values were measured in triplicate by assessing turbidity (OD600). The absence of turbidity (OD600 < 0.1) was interpreted as growth inhibition.

### Gene set enrichment analysis (GSEA)

Gene set enrichment analysis for transcriptomics and proteomics data was performed using the COG 2024 (Galperin et al., 2025) and VFDB (Zhou et al., 2025) databases. The analysis and visualization were conducted using the DGE-ontology 1.0 library (Bukowski and Wladyka, 2024). The mapping of COG and VFDB categories to *S. aureus* 3956/13/K transcripts and protein products is provided in the Supplementary material: USA300K_metadata.tsv.

### Analysis of PemK-Sa1 target motif distribution

The relationship between UAUU motif density and differential expression was assessed across all transcripts at two levels. First, the association between UAUU density (counts per 100 nt) and log_2_ fold change (LFC) was quantified by Pearson and Spearman correlation, the latter to capture monotonic but non-linear trends without distributional assumptions. Second, to model motif occurrence directly while accounting for transcript length, raw UAUU counts were fitted with a negative-binomial generalized linear model (log link) using log transcript length as an offset, so that coefficients represent log rate-ratios of motif density per nucleotide. The negative-binomial family was used in preference to Poisson to accommodate over-dispersion, with the dispersion parameter *α* estimated by maximum likelihood. The model was fitted with continuous LFC as the predictor across all transcripts (*n* = 2,604), and overall significance assessed by likelihood-ratio test against the intercept-only model. As an interpretable illustration of the effect, the same model was additionally fitted to the robustly regulated transcripts only (down, LFC ≤−1; up, LFC ≥ 1; *n* = 950) with up-regulated transcripts as the reference level. General data analysis was carried out in JupyterLab (4.5) using Python (3.12), NumPy (2.4) and Polars (1.39) libraries (Harris et al., 2020). Correlations were computed with SciPy (1.17; pearsonr, spearmanr) and GLMs with Statsmodels (0.14; negativebinomial) libraries (Virtanen et al., 2020; Seabold and Perktold, 2010). Reported confidence intervals are the model-based 95% intervals, exponentiated to the rate-ratio scale. Visualizations were prepared using Matplotlib (3.10) and Seaborn (0.13) libraries (Hunter, 2007; Waskom, 2021).

## Results

### PemK-Sa1 toxin reduces the antibiotic resistance of multidrug-resistant *Staphylococcus aureus*

**Figure 1:**
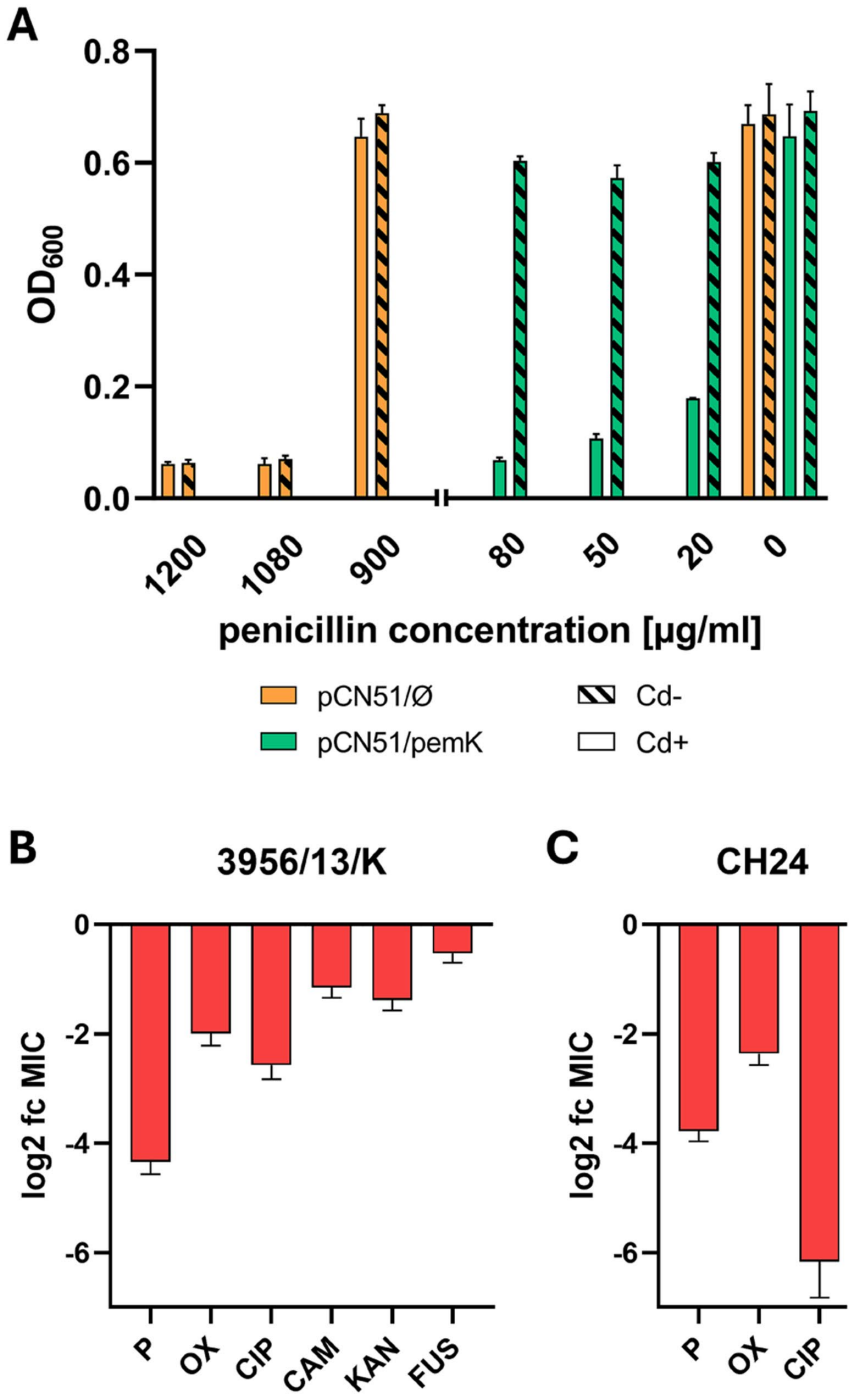
PemK-Sa1 expression decreases resistance to antibiotics. **(A)** Growth, monitored by *OD*600, of *S. aureus* 3956/13/K/ carrying PemK-Sa1-expressing or a control (Ø) plasmid in presence of penicillin. Minimal inhibitory concentration (MIC) is defined as the lowest antibiotic concentration inhibiting bacterial growth below *OD*600 of 0.1. **(B,C)** Fold change of MIC for antibiotics of PemK-Sa1-expressing *S. aureus* strains (3,956/13/K, and CH24, respectively) relative to the strains carrying an empty plasmid. P, penicillin; OX, oxacillin; CIP, ciprofloxacin; CAM, chloramphenicol; KAN, kanamycin; FUS, fusidic acid.

We recently demonstrated that type II TA systems exhibit a negative correlation with several antibiotic resistance determinants in *S. aureus*. Moreover, the PemK-Sa1 toxin decreases resistance to chloramphenicol (Bukowski et al., 2025). Here, we asked whether the toxin exerts a similar effect on the resistance to other antibiotics. To this end, we chose two multidrug-resistant *S. aureus* strains and determined their susceptibility/resistance to selected antibiotics. The 3956/13/K strain was isolated from a human patient and belongs to the highly successful USA300 lineage of community-associated methicillin-resistant *S. aureus* (Carrel et al., 2015). The complete genome of the strain consisted of a 2,901,849 bp chromosome and three plasmids: pBU108a (34,810 bp), pAH5667 (13,163 bp), and pUSA395613K (4,655 bp). The chromosome showed a GC content of 32.80%, contained 2,804 protein-coding sequences and 82 RNA genes. Based on in silico analysis, the strain was identified as MLST ST8, which belongs to clonal complex (CC) 8, and spa type t13053. The search for SCC*mec* using the SCCmecFinder identified an SCC*mec* IV(2B) structure. The second strain, CH24, is an MRSA isolated from a chicken and genotypically related to poultry-origin strains naturally carrying the PemIK-Sa1 TA system (Lowder et al., 2009; Polakowska et al., 2012). However, neither 3956/13/K nor CH24 naturally carries the PemIK-Sa1 TA system. Both bacteria were resistant to β-lactam antibiotics (penicillin, ampicillin, oxacillin, and cefoxitin), which agrees with the presence of the staphylococcal cassette chromosome *mec* (SCC*mec*) carrying *mecA* in their genomes. Moreover, 3,956/13/K was resistant to ciprofloxacin, kanamycin, fusidic acid, and chloramphenicol. Both strains were susceptible to tetracycline and erythromycin. This enabled us to transform the strains with a pCN51-based plasmid carrying the coding sequence for PemK-Sa1 (pCN51-pemKSa1) and the intact PemIK-Sa1 system under the control of a cadmium-inducible promoter. Then, we determined MIC values for the selected antibiotics for the strains expressing the PemK-Sa1 toxin and for the control (carrying an empty pCN51, 0.5 μM Cd^2+^, as an inducer, was present in both strains) (Table 1, Supplementary Table S1). EUCAST and CLSI-recommended microdilution assays revealed that in *S. aureus* 3956/13/K expressing the PemK-Sa1 toxin, in comparison to the control variant, the MIC value was decreased 20 times for penicillin (from 1,050 to 52 μg/mL; Figure 1A), nearly six times for ciprofloxacin (from 77 to 13 μg/mL), four times for oxacillin (from 302 to 76 μg/mL), almost three times for kanamycin (from 288 to 111 μg/mL), and over two times for chloramphenicol (from 73 to 33 μg/mL). Of note, expression of the PemK-Sa1 toxin does not significantly alter fusidic acid resistance (from 43 to 30 μg/mL). Similarly, for the strain *S. aureus* CH24, the decrease of MIC values was 14 times for penicillin (from 1,100 to 80 μg/mL), over 70 times for ciprofloxacin (from 15 to 0.21 μg/mL), and five times for oxacillin (from 36 to 7 μg/mL). This clearly shows that the expression of PemK-Sa1, mimicking activation of the whole TA system, substantially decreases resistance to almost all tested antibiotics (Figures 1B,C).

**Table 1:** MIC values for *S. aureus* strains transformed with a plasmid expressing intact PemIK-Sa1 TA system, PemK-Sa1 toxin, or carrying a control plasmid (Ø).

Antibiotic	Strain	MIC [μg/mL]
Penicillin	3956/13/K-Ø	1,050 ± 260
	3956/13/K-pemK	52 ± 10
	3956/13/K-pemIK	1,067 ± 58
	CH24-Ø	1,100 ± 173
	CH24-pemK	80 ± 10
	CH24-pemIK	1,017 ± 161
Oxacillin	3956/13/K-Ø	302 ± 7
	3956/13/K-pemK	76 ± 3
	3956/13/K-pemIK	294 ± 18
	CH24-Ø	36 ± 0
	CH24-pemK	7 ± 1
	CH24-pemIK	30 ± 6
Ciprofloxacin	3956/13/K-Ø	77 ± 6
	3956/13/K-pemK	13 ± 6
	3956/13/K-pemIK	130 ± 0
	CH24-Ø	15 ± 6
	CH24-pemK	0.21 ± 0.09
	CH24-pemIK	12 ± 0
Chloramphenicol^*^	3956/13/K-Ø	73 ± 6
	3956/13/K-pemK	33 ± 6
	3956/13/K-pemIK	70 ± 0
Kanamycin^*^	3956/13/K-Ø	288 ± 41
	3956/13/K-pemK	111 ± 22
	3956/13/K-pemIK	317 ± 35
Fusidic acid^*^	3956/13/K-Ø	43 ± 5
	3956/13/K-pemK	30 ± 0
	3956/13/K-pemIK	42 ± 4.4

*determinants not present in the CH24 strain (susceptible).

Expression was induced by 0.5 μM CdCl_2_.

### PemK-Sa1 toxin affects the expression of one-third of the *Staphylococcus aureus* transcriptome

**Figure 2:**
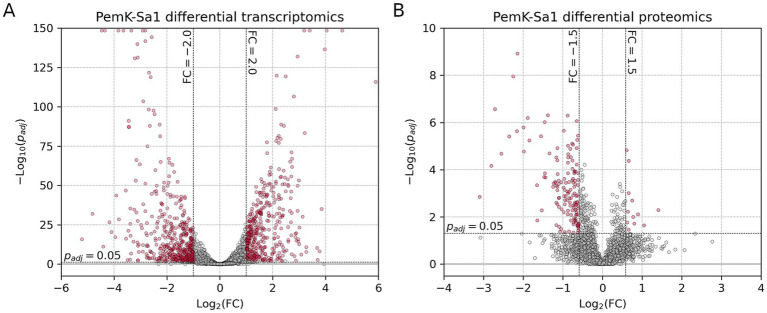
PemK-Sa1 expression causes significant changes in the *S. aureus* transcriptome and proteome. **(A)** Volcano plot of differential transcriptomics illustrating bidirectional changes in gene expression. Non-significantly and significantly differentiating transcripts (fold change (FC) ≥ 2, *pad_j_* < 0.05) are marked with grey and red circles, respectively. **(B)** At the proteome level, the volcano plot shows that PemK-Sa1 expression mainly results in protein downregulation. Non-significantly and significantly differentiating proteins (fold change (FC) ≥ 1.5, *pad_j_* < 0.05) are marked with grey and red circles, respectively.

**Figure 3:**
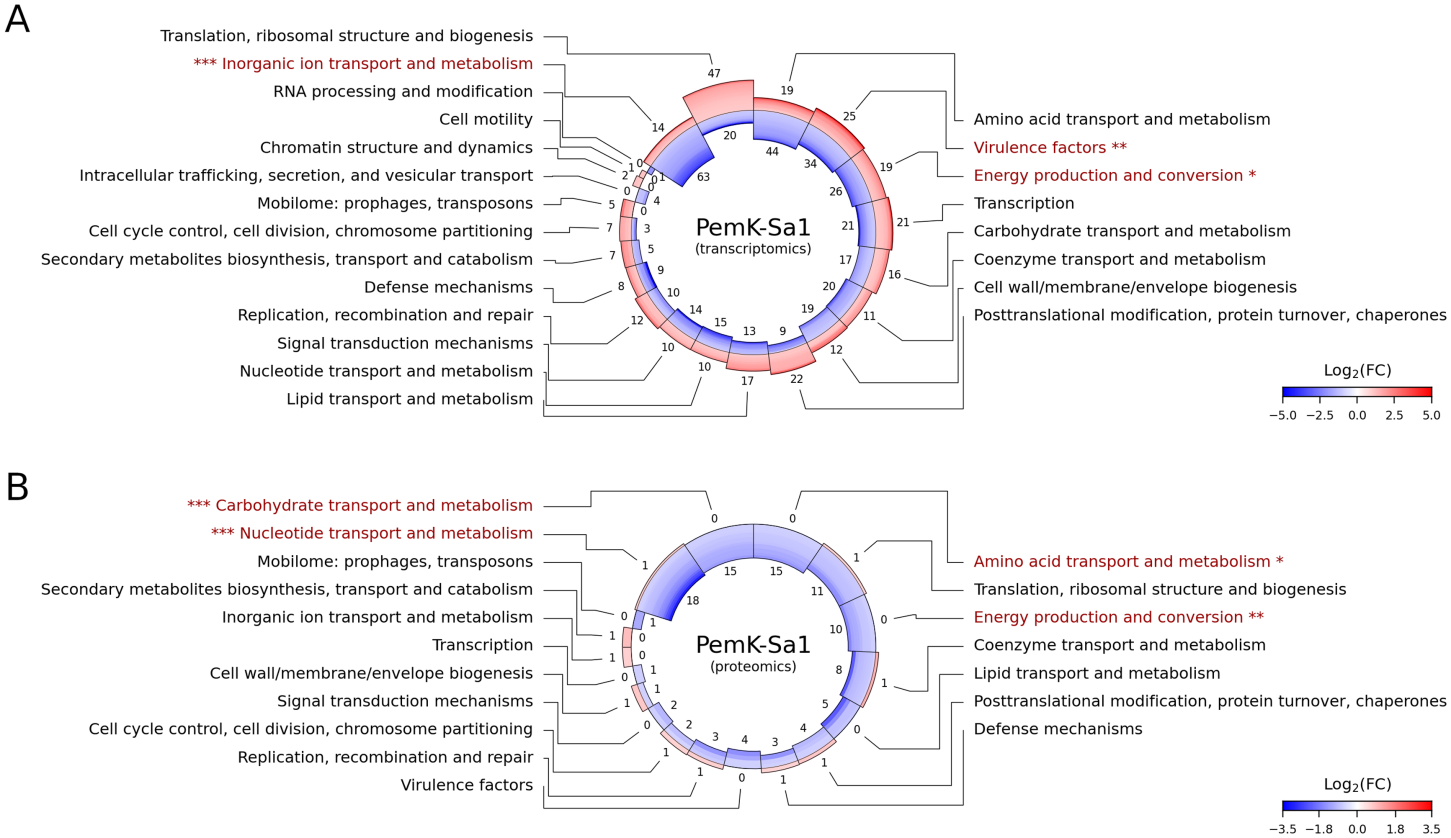
Gene set enrichment analysis of PemK-Sa1 expression on the transcriptome **(A)** and proteome **(B)** levels. **(A)** In transcriptomics, three groups of genes were significantly affected; specifically, transcripts for proteins involved in inorganic ion transport and metabolism were mainly downregulated. **(B)** In proteomics, all four protein groups significantly affected by the toxin were almost exclusively downregulated. *, **, and *** indicate hypergeometric distribution probabilities of 0.05, 0.01, and 0.001, respectively.

Since PemK-Sa1 decreases antibiotic resistance to β-lactams, fluoroquinolones, aminoglycosides, and chloramphenicol, we asked for the basis of this phenomenon. First, we applied a transcriptomic approach to assess and quantify transcript levels of determinants directly involved in antibiotic resistance and other genes. *S. aureus* 3956/13/K, transformed with pCN51/Ø or pCN51/pemKSa1, was grown to the logarithmic phase, and the expression of toxins was induced with cadmium. Forty-five minutes after induction, RNA was collected and subjected to RNA-Seq. Differential gene expression (DGE) analysis revealed that 442 and 517 gene transcripts were up- and down-regulated by PemK-Sa1 toxin (≥2-fold, *p* < 0.05), respectively, encompassing one-third of the transcriptome (Figure 2A, Supplementary Figure S1). 10 the most upregulated transcripts (>10-fold change) by PemK-Sa1, among others, code for coagulase (*coa*), immunodominant staphylococcal antigen IsaB (*isaB*), myeloperoxidase inhibitor SPIN, and fibronectin-binding protein A (*fnbA*). Among 61 transcripts highly upregulated (5-10-fold change) by the toxin expression were those coding for chaperone GroES (*groES*), fibronectin-binding protein B (*fnbB*), clumping factor A (*clfA*), fibrinogen-binding protein (*ecb*), and tRNAs for 11 amino acids (S, E, K, L, M, R, H, D, T, W, and G) (Supplementary Table S2). However, tRNAs for the remaining amino acids were also significantly upregulated. This is likely related to the scarcity of UAUU motifs in tRNAs. When the bulk of mRNA is degraded by PemK-Sa1, transcripts that escape cleavage appear enriched after normalization. As a result, the surviving tRNAs could continue to support translation of UAUU-poor transcripts. This may explain why the PemK-Sa1 toxin leads to relatively higher levels of UAUU-poor transcripts, such as those of many staphylococcal virulence factors, and experimentally confirms our earlier predictions (Bukowski et al., 2013). Interestingly, even among 371 moderately upregulated (2-5-fold change) transcripts, we did not identify any coding for determinants directly involved in antibiotic resistance. Thus, PemK-Sa1 expression does not result in significant upregulation of antibiotic resistance genes in the *S. aureus* transcriptome (for details, see the chapter below). Analysis of PemK-Sa1-driven downregulated transcripts revealed 25 genes with >10-fold change and 71 and 421 genes with high and moderate downregulation, respectively. Among the most downregulated transcripts were those coding for the Agr system (*agrA-D*), chemotaxis inhibitory protein (*chp*), acyl-CoA dehydrogenase (*caiA*), lantibiotic ABC transporter (ACMTNW_13470-ACMTNW_13475), other ABC transporters (*ssuABC*, *pmtA-D*), and multiprotein Na+/H+ antiporter (*mnhA-G*). Highly downregulated transcripts also coded for two-component systems, transmembrane transporters, and gene expression regulatory proteins. Moreover, many genes encoding proteins involved in basic metabolism and energy production were downregulated (Supplementary Table S2). Among the downregulated transcripts, we identified several genes encoding antibiotic resistance determinants, which are described in detail in the following section. To holistically assess the impact of PemK-Sa1 on gene expression, we assigned ontologies to all genes and performed gene set enrichment analysis (GSEA) (Supplementary Table S2). As a result, we identified three functional groups of genes, namely, (i) inorganic ion transport and metabolism, (ii) virulence factors, and (iii) energy production and conversion, whose expression is significantly affected by the toxin (Figure 3A). The influence of PemK-Sa1 on a large number of genes responsible for processes vital to the cell explains the observed growth inhibition upon induction of the toxin’s expression (Supplementary Figure S2).

**Figure 4:**
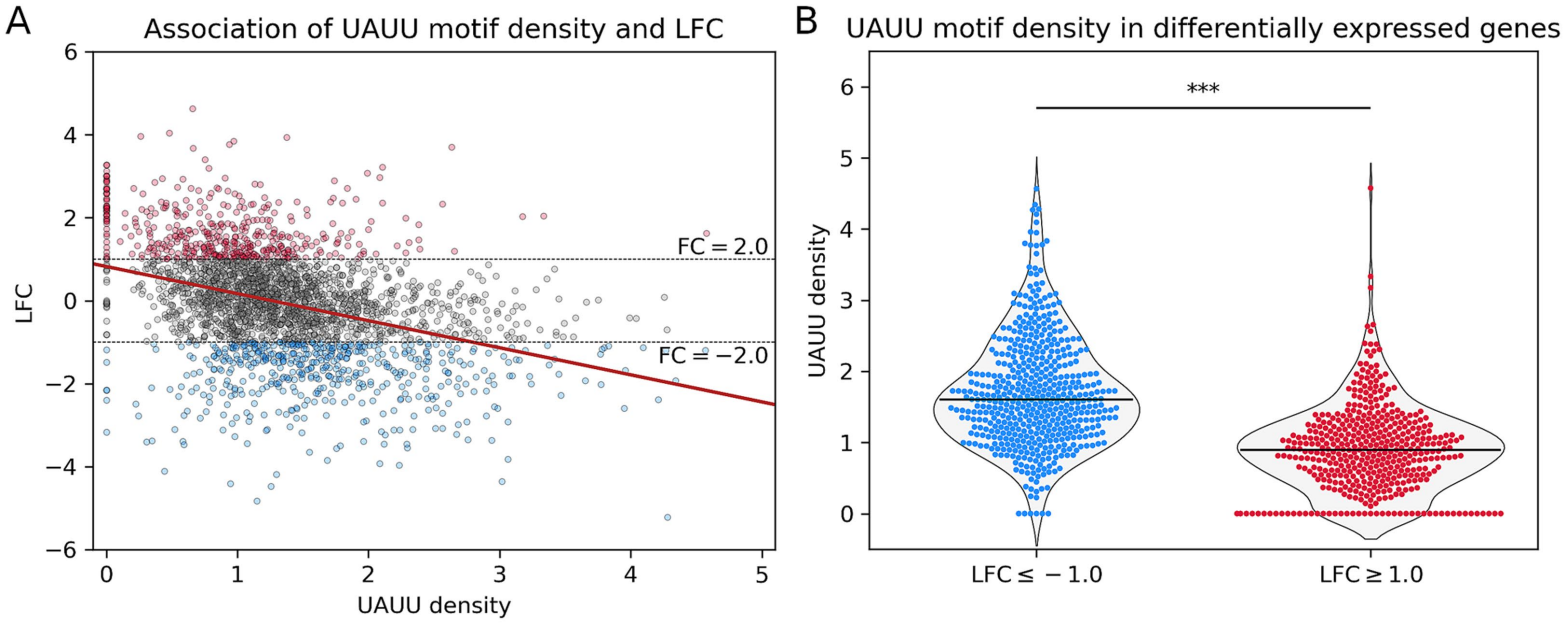
UAUU motif density is inversely associated with transcript regulation by PemK-Sa1 toxin. **(A)** Log_2_ fold change (*LFC*) plotted against UAUU motif density (counts per 100 nt) for all transcripts. Each point is one transcript: Up-regulated (*LFC* ≥ 1, red), down-regulated (*LFC* ≤ −1, blue), and unchanged (gray); dashed lines mark the fold thresholds. The red line is the linear regression fit. UAUU density and *LFC* are negatively correlated (Pearson *r* = −0.368, *p* = 2.47e-84; Spearman *ρ* = −0.393, *p* = 1.05e-96). **(B)** UAUU density in the robustly regulated groups of genes (down, *LFC* ≤ −1; up, *LFC* ≥ 1). Black lines indicate group medians. Down-regulated transcripts carry 1.71-fold higher UAUU density than up-regulated transcripts (negative-binomial GLM, ***p < 0.001).

UAUU motif density was inversely associated with transcript regulation: more strongly up-regulated transcripts carried progressively less UAUU per nucleotide. Across all transcripts, UAUU density (counts per 100 nt) correlated negatively with log_2_ fold change (LFC) by both Pearson (r = −0.368, *p* = 2.47e-84) and Spearman (*ρ* = −0.393, *p* = 1.05e-96) correlation, the close agreement of the two indicating a consistent, largely monotonic trend (Figure 4A). Modeling raw UAUU counts directly with a negative-binomial GLM and a transcript-length offset confirmed this relationship while accounting for length: density decreased by a factor of 0.86 per unit increase in LFC (rate ratio 0.86, 95% CI 0.85–0.88; *n* = 2,604; likelihood-ratio *p* = 1.945e-85). Contrasting the robustly regulated groups of genes directly (down, LFC ≤ −1; up, LFC ≥ 1), down-regulated transcripts carried 1.71-fold higher UAUU density than up-regulated transcripts (95% CI 1.60–1.81; *n* = 950; *p* = 1.025e-58) (Figure 4B). As expected for a single sequence feature under multifactorial regulation, UAUU density explained only a small fraction of the variation (McFadden’s pseudo-*R*^2^ = 0.026 and 0.047 for the continuous and categorical models, respectively). Nevertheless, the association was highly robust and directionally consistent across all four analyses. Together these results indicate that UAUU-rich transcripts skew toward down-regulation, a pattern consistent with UAUU being the PemK-Sa1 target.

### PemK-Sa1 toxin decreases the expression of genes directly involved in antibiotic resistance

The decreased resistance to antibiotics in PemK-Sa1-expressing strains may result from reduced fitness due to altered expression of one-third of genes. However, a direct influence of the toxin on the expression of antibiotic resistance determinants is even more plausible. Therefore, we used the Resistance Gene Identifier (RGI) in the Comprehensive Antibiotic Resistance Database (CARD) to identify genes associated with antibiotic resistance in *S. aureus* 3956/13/K. We have identified 23 AR determinants and quantified their transcript level in the strain expressing PemK-Sa1 toxin compared to the control (3956/13/K-Ø) using the same RNA-seq/DGE dataset described in the above chapter (Table 2). Cadmium ions were added to both strains 45 min prior to RNA collection to exclude the nonspecific effect of the inducer on gene expression in DGE analysis. Expression of four genes (*norB*, *norC*, *mepA*, and *abcA*) encoding efflux pumps involved in fluoroquinolones and oxacillin resistance was decreased in 3956/13/K/-pemKSa1. Moreover, genes (*norG*, *arlS*, and *mgrA*) coding for proteins directly controlling the expression of these efflux pumps were also downregulated. The gene *aacA-aphD* encoding aminoglycoside acetyltransferase(6′)-Ie/aminoglycoside phosphotransferase(2″)-Ia, a bifunctional enzyme that is the major source of aminoglycoside resistance, was substantially (3.9-fold) downregulated. The *mecA* gene, which confers resistance to *β*-lactams, was downregulated slightly (1.4-fold). However, the transcript level for *mecR1*, which encodes a sensor for β-lactams, was 2.5-fold decreased. The plasmid-encoded cluster encoding resistance to penicillin was also downregulated (*blaZ* – 1.4-fold, *blaR1*–1.4-fold, and *blaI* – 2.5-fold). The *fusA*, which encodes elongation factor G and carries two point mutations conferring resistance to fusidic acid, was not affected by PemK-Sa1. Neither *norA* nor *lmrS*, encoding efflux pumps involved in resistance to ciprofloxacin and chloramphenicol, respectively, was differentially expressed. The *cam* transcript, encoding chloramphenicol acetyltransferase, was also unaltered. Interestingly, the *fosB*, encoding bacillithiol S-transferase responsible for resistance to fosfomycin was transcriptionally silent, which agrees with phenotypic susceptibility to this antibiotic. Two genes, *ermC* and *blaT*, were delivered via the shuttle vector pCN51, conferring erythromycin resistance in *S. aureus* and ampicillin resistance in *E. coli*. The genes were down-regulated (2.7-fold) and up-regulated (3.1-fold), respectively.

**Table 2:** Differential gene expression of genes providing resistance to antibiotics in *S. aureus* 3956/13/K following expression of PemK-Sa1 toxin.

Locus tag (UAUU count / freq)	Gene	Product	PemK-Sa1-induced fold change	Resistance
ACMTNW_00160 (24/1.2)	*mecA*	PBP2a family beta-lactam-resistant peptidoglycan transpeptidase MecA	−1.42	penicillin beta-lactam antibiotic
ACMTNW_00575 (33/2.4)	*norC*	multidrug efflux MFS transporter NorC	−3.01	fluoroquinolone antibiotic; disinfecting agents and antiseptics
ACMTNW_01790 (7/1.7)	*mepR*	multidrug efflux transporter transcriptional repressor MepR	1.54	glycylcycline; tetracycline antibiotic
ACMTNW_01795 (33/2.4)	*mepA*	multidrug efflux MATE transporter MepA	−3.71	glycylcycline; tetracycline antibiotic
ACMTNW_03505 (30/1.7)	*abcA*	multidrug efflux ABC transporter	−2.74	peptide antibiotic, penicillin beta-lactam antibiotic, cephalosporin, moenomycin antibiotic
ACMTNW_03730 (5/1.1)	*mgrA*	HTH-type transcriptional regulator MgrA	−5.54	fluoroquinolone antibiotic; cephalosporin; penicillin beta-lactam antibiotic; tetracycline antibiotic; peptide antibiotic; moenomycin antibiotic; disinfecting agents and antiseptics
ACMTNW_03775 (25/2.1)	*norA*	multidrug efflux MFS transporter NorA	1.46	fluoroquinolone antibiotic
ACMTNW_07155 (22/1.6)	*arlS*	sensor histidine kinase ArlS	−2.60	fluoroquinolone antibiotic; disinfecting agents and antiseptics
ACMTNW_07160 (5/0.8)	*arlR*	response regulator transcription factor ArlR	−1.03	fluoroquinolone antibiotic; disinfecting agents and antiseptics
ACMTNW_07275 (30/2.2)	*norB*	multidrug efflux MFS transporter NorB	−3.03	fluoroquinolone antibiotic; disinfecting agents and antiseptics
ACMTNW_08815 (29/2.0)	*aph(2″)-Ia*	aminoglycoside O-phosphotransferase APH(2″)-Ia	−3.85	aminoglycoside antibiotic
ACMTNW_11165 (37/1.4)	*kdpD*	DUF4118 domain-containing protein	−1.89	aminoglycoside antibiotic
ACMTNW_11660 (18/1.2)	*lmrS*	multidrug efflux MFS transporter LmrS	1.47	macrolide antibiotic; aminoglycoside antibiotic; oxazolidinone antibiotic; diaminopyrimidine antibiotic; phenicol antibiotic
ACMTNW_11665 (13/2.7)	*sepA*	multidrug efflux transporter SepA	−4.00	disinfecting agents and antiseptics
ACMTNW_11670 (25/1.9)	*sdrM*	multidrug efflux MFS transporter SdrM	−1.34	fluoroquinolone antibiotic; disinfecting agents and antiseptics
ACMTNW_12520 (8/1.9)	*fosB*	bacillithiol S-transferase	N/A (transcriptionally silent)	phosphonic acid antibiotic
ACMTNW_00030 (28/1.1)	*gyrA* (S84L)	DNA gyrase subunit A	−1.34	fluoroquinolone antibiotic
ACMTNW_02900 (10/0.5)	*fusA* (H457Q, L461F)	elongation factor G	−1.28	fusidane antibiotic
ACMTNW_06855 (37/1.5)	*parC* (S80Y)	DNA topoisomerase IV subunit A	−1.45	fluoroquinolone antibiotic
ACMTNW_14825 (9/1.1)	*blaZ*	penicillin-hydrolyzing class A beta-lactamase BlaZ	−1.37	penicillin beta-lactam antibiotic
ACMTNW_14855 (9/1.4)	*catA*	type A-7 chloramphenicol O-acetyltransferase	1.66	phenicol antibiotic
pCN51_01 (11/1.5)	*ermC*	adenine methylase ErmC	−2.66	macrolide antibiotic
pCN51_02 (7/0.8)	*blaT*	beta-lactamase TEM-116	3.15	penicillin beta-lactam antibiotic (in *E. coli*)

### Proteomics reveals that the PemK-Sa1 toxin impacts key metabolic traits

PemK-Sa1 toxin has a severe impact on gene expression, as shown by transcriptomic analysis. Therefore, we asked whether it was reflected in the protein level. To this end, we isolated cellular proteins 90 min after toxin expression was induced and performed a 2D DIGE analysis (Supplementary Figure S3). Protein spots that differed between samples expressing toxin (3956/13/K-pemKSa1) and the control (3956/13/K-Ø) were cut out from the gel and identified by mass spectrometry. Expression of PemK-Sa1 affects the expression of 108 proteins, among which 96 and 12 were down- and upregulated, respectively (Figure 2B; Supplementary Table S3). Among the proteins the most downregulated by PemK-Sa1 were bifunctional phosphoribosylaminoimidazolecarboxamide formyltransferase/inosine monophosphate cyclohydrolase (PurH), formate--tetrahydrofolate ligase (Fhs), and IMP dehydrogenase (GuaB), all involved in nucleotide transport and metabolism. The two most upregulated proteins were pyridoxamine 5′-phosphate oxidase (ACMTNW_12770) and hydroxymethylpyrimidine/phosphomethylpyrimidine kinase (ThiD), involved in vitamin B_1_ synthesis. Proteomic changes correspond well with the transcriptomic data. Sixty eight differentiating proteins (63.0%) were regulated in the same direction as their respective transcripts. Among the remaining 40, transcripts for only 8 were strongly (>2-fold) inversely regulated (7.4%) (Supplementary Figure S4). The GSEA shows that PemK-Sa1-affected proteins belong to four functional groups involved in nucleotide, amino acid, and carbohydrate transport and metabolism, as well as energy production and conversion (Figure 3B). Specifically, six enzymes (Pgi, TpiA, GapA, GpmA, Eno, and PykA) out of 10 involved in glycolysis were downregulated in bacteria expressing the PemK-Sa1 toxin. Moreover, enzymes involved in the *de novo* synthesis of purine nucleotides, encoded in the *pur* operon, were also downregulated. Furthermore, α- and β-subunits of ATP synthase (AtpA and AtpD, respectively) were downregulated. Decreased levels of a number of proteins implicated in vital cellular processes reflect not only impaired growth (Supplementary Figure S2) but also could be directly linked to reduced resistance to various antibiotics.

## Discussion

The growing resistance of bacterial pathogens to antibiotics is an emerging problem. Methicillin-resistant *S. aureus* (MRSA) is on the World Health Organization Bacterial Priority Pathogen List (Sati et al., 2025; WHO, 2024). TA systems were shown to be implicated in the maintenance of mobile genetic elements (MGEs), which often carry antibiotic resistance (AR) determinants and, as such, are linked with the stabilization and spread of drug resistance among bacteria (Huguet et al., 2016; Ramamurthy et al., 2022). In contrast, our recent analysis shows a negative correlation between the co-occurrence of AR determinants and TA systems, particularly those involving toxins with ribonucleolytic activity (Bukowski et al., 2025). However, the mechanisms behind this finding have not been experimentally verified.

Here, we confirmed that the PemK-Sa1 toxin from the PemIK-Sa1 TA system significantly decreases antibiotic resistance in MRSA strains. The reduction in resistance for strains expressing the toxin ranges from 2-fold for chloramphenicol in 3956/13/K to over 70-fold for ciprofloxacin in CH24. Interestingly, according to CLSI guidelines, the decrease in MIC for the latter (from 15.0 to 0.21 μg/mL) makes the CH24-pemKSa1 strain sensitive to this antibiotic (Lewis et al., 2024). Although such a significant reduction in resistance was observed only for a single toxin, antibiotic, and strain, this suggests that activation of TA systems may be relevant to medical practice. The application of toxins from TA systems, either directly or through artificial activation of the systems, has been suggested as a way to kill bacteria or inhibit their growth (Williams and Hergenrother, 2012). The toxicity and metabolic burden caused by the toxins are analogous to the action of antibiotics. In such an approach, the toxins may be considered an alternative to these drugs for the treatment of antibiotic-resistant pathogens (López-Igual et al., 2019; Równicki et al., 2020). Our results indicate that such toxins may also restore the efficacy of antibiotics against such bacteria.

PemK-Sa1 decreases antibiotic resistance against different groups: β-lactams, fluoroquinolones, aminoglycosides, and amphenicols. Each group has different molecular targets and affects processes vital for bacteria. β-lactam antibiotics bind to enzymes crosslinking peptidoglycan and block the synthesis of bacterial cell walls. Fluoroquinolones stop DNA replication by inhibiting gyrase and topoisomerase IV, whereas aminoglycosides and amphenicols disrupt protein synthesis by binding to 30S and 50S subunits of the bacterial ribosome, respectively (Hooper and Jacoby, 2016; Krause et al., 2016; Lin et al., 2018). Resistance to antibiotics is conferred by various mechanisms, including modification of molecular targets, degradation of antibiotics, or decrease of their intracellular concentrations by efflux pumps and ABC transporters. Moreover, the expression of determinants conferring resistance to antibiotics may be controlled by dedicated and/or global regulators (Depardieu et al., 2007; Dersch et al., 2017; Du et al., 2018). Therefore, the level of resistance, which is routinely quantified by MIC, depends not only on the presence of particular determinants but also on their possible redundancy and other indirect regulatory factors.

Resistance to ciprofloxacin, a representative of fluoroquinolones, is conferred by efflux pumps NorA, NorB, NorC, and MepA (Hooper and Jacoby, 2015). Except for *norA*, the genes for the other pumps were downregulated by PemK-Sa1. The second mechanism responsible for resistance to this group of antibiotics is based on single-nucleotide polymorphisms of genes encoding subunit A of gyrase (GyrA) and topoisomerase IVa (ParC) (Fàbrega et al., 2009; Tanaka et al., 2000). In *S. aureus* 3956/13/K *parC*, a TCC → TAC mutation in codon 80 confers resistance to ciprofloxacin. The *parC* transcript level is only slightly (1.4 times) downregulated by PemK-Sa1. The presence of five determinants conferring resistance to ciprofloxacin results in a high MIC value for this strain. Our transcriptomic data show that three of them are significantly downregulated by PemK-Sa1, which would explain the observed six-fold decrease in resistance. Interestingly, CH24 carries only three resistance determinants (NorA, NorB, and NorC) for this antibiotic. This is reflected in the lower MIC value. Moreover, assuming a similar downregulation of *norB* and *norC* by the toxin, as experimentally shown for 3956/13/K, we suspect that the loss of resistance to ciprofloxacin in CH24 results from direct degradation of transcripts encoding the majority of resistance determinants to this antibiotic.

In MRSA strains, resistance to β-lactams is driven by *mecA*, which encodes a variant of penicillin-binding protein (PBP2a) that is not inhibited by the antibiotics (Hartman and Tomasz, 1984). Moreover, penicillin-resistant strains produce a β-lactamase (BlaZ), which degrades penicillin and its derivatives. However, such an enzyme does not decompose methicillin and its homologs, such as oxacillin (Lade and Kim, 2023). Resistance to β-lactam antibiotics in PemK-Sa1-expressing strains was significantly decreased. Interestingly, the MIC reduction was higher for penicillin (20-fold) than for oxacillin (4-fold). Higher susceptibility to penicillin could be attributed to the decrease of *blaZ* (1.4-fold), *blaR1* (1.4-fold), and *blaI* (2.5-fold) in the toxin-expressing strains. The *mecA* gene, which drives resistance to both *β*-lactams, was also slightly decreased (1.4-fold). However, the *mecR1* gene was more strongly affected (2.5-fold) by the toxin. The latter is responsible for sensing antibiotics, leading to increased expression of *mecA* and an adaptive increase in resistance to β-lactams (Alexander et al., 2023; Hackbarth and Chambers, 1993). Downregulation of *mecR1* impairs the expression of *mecA* and, as a consequence, decreases resistance to these antibiotics. The effect, however, is not clearly observed in our transcriptomic assay because the induction of PemK-Sa1 expression occurred in a medium without β-lactam antibiotic.

The decrease in resistance to chloramphenicol upon induction of PemK-Sa1 expression was only twofold. In *S. aureus*, the resistance to this antibiotic is conferred either by chloramphenicol acetyltransferase, Cat, which inactivates the drug, or by the major facilitator superfamily (MFS) transporter, LmrS, which is a multidrug efflux pump (Floyd et al., 2010). It was postulated that the decrease in resistance to chloramphenicol in the presence of PemK-Sa1 is primarily caused by the toxin cleaving the *cat194* gene transcript (Bukowski et al., 2025). Our transcriptomic data show that the expression of both genes was not significantly affected by PemK-Sa1. This apparent discrepancy may be explained by the two different genes used in these studies. Although the encoded acetyltransferases share 54% identity, the genes have no significant similarity. Moreover, the *cam* transcript contains fewer toxin-target sequences (UAUU) than *cat194* (8 vs. 13, respectively). Therefore, the decreased resistance to chloramphenicol in *S. aureus* 3956/13/K is not the direct result of the degradation of antibiotic resistance determinants transcript but rather secondary events resulting from RNase toxin activity toward other targets.

Interestingly, the expression of PemK-Sa1 toxin does not increase susceptibility to fusidic acid. Resistance to this antibiotic is conferred by point mutations in *fusA* (Besier et al., 2003; Castanheira et al., 2010), which encodes elongation factor G, an indispensable component of protein synthesis. Mechanisms of resistance to other antibiotics (β-lactams, fluoroquinolones, aminoglycosides, and chloramphenicol) used in the study are based on the presence of additional genes encoding enzymes or other proteins that degrade, modify, or pump out antibiotics, or that replace/support drug targets. Their respective transcripts are downregulated during PemK-Sa1 expression, which could be linked with decreased antibiotic resistance. In contrast, resistance to fusidic acid in *S. aureus* 3956/13/K is conferred by two subtle mutations in the essential core genome gene that do not change the number of PemK-Sa1 target sequences (10; UAUU) in the gene transcript. It was shown that the target sequence for the toxin is not randomly distributed in the *S. aureus* transcriptome and that the toxin does not inhibit protein synthesis (Bukowski et al., 2013). Indeed, *fusA* belongs to a set of genes where the PemK-Sa1 target sequence is underrepresented. This suggests that the gene, regardless of whether it confers resistance to fusidic acid, escapes the toxin’s action.

Expression of PemK-Sa1 affects one-third of the transcriptome (959/2839; 33.8%). Although the toxin is an RNase, the number of down- and upregulated transcripts is comparable (517 vs. 442). Nevertheless, regardless of the direction of change, the toxin-induced burden justifies the observed bacterial growth inhibition and may affect antibiotic resistance. Based on GSEA, a few functional gene groups were identified as strongly affected by PemK-Sa1 expression. Genes coding for proteins involved in inorganic ions transport and metabolism were mostly downregulated. Among them were *mnhA-G1* and *mnhA-G2* operons encoding secondary antiporters that catalyze K^+^ and/or Na^+^ ion efflux in exchange for H^+^ ions. Mnh1 and Mnh2 have essential roles in halotolerance and osmotolerance, respectively. Moreover, deleting the *mnhA1* gene led to a significant loss of *S. aureus* virulence in a mouse model (Vaish et al., 2018). The second highly downregulated operon (*pmtA-D*) encodes an ABC transporter responsible for the export of phenol-soluble modulins (PSMs). These highly cytotoxic peptides are important virulence factors and bacteriocins. In the absence of *pmt*, cytosolic accumulation of PSMs was observed, resulting in abnormal cell division and severe damage to the cytoplasmic membrane (Chatterjee et al., 2013), which may affect susceptibility to antibiotics.

The expression of the PemK-Sa1 toxin resulted in the downregulation of most genes in the *pur* operon, as evidenced by transcriptomics and proteomics, suggesting an impediment to nucleotide biosynthesis. *De novo* purine biosynthesis is a significant pillar of nucleotide metabolism, crucial for cell growth via nucleic acids and ATP synthesis (Li et al., 2018). *Staphylococcus aureus* strains with *purB*, *purF*, *purH*, and *purM* mutations showed defective persistence in multiple stress conditions, including antibiotics such as rifampicin and gentamicin (Yee et al., 2015). Similarly, Δ*purN* exhibited increased susceptibility to *β*-lactam and fluoroquinolone antibiotics, especially during exponential growth (Peng et al., 2022). Moreover, the *purF* mutation rendered the bacterium highly susceptible to vancomycin treatment (Li et al., 2018). The demand for ATP has recently been linked to antibiotic susceptibility (Yang et al., 2019). Moreover, the ATP synthase complex has been postulated as an important determinant of intrinsic resistance to aminoglycoside antibiotics in *S. aureus*. Deleting genes from the *atp* operon resulted in a 16-fold increase in susceptibility to gentamicin (Vestergaard et al., 2016, 2017). In our study, *atpA* and *atpD*, which encode the ATP synthase subunits *α* and β directly involved in ATP generation, were significantly downregulated at both the transcript and protein levels. This is consistent with the decreased resistance to kanamycin observed in the strain expressing the PemK-Sa1 toxin and may serve as a parallel pathway for the direct degradation of the *aacA-aphD* gene transcript.

Considering the study’s limitations, we must note that the toxic effects and decreased resistance to antibiotics were due to the artificial expression of the PemK-Sa1 toxin, not to the entire PemIK-Sa1 TA system. Such an imbalanced expression of TA system components may overscore the role of the toxin. On the other hand, factors that activate PemIK-Sa1 are currently unknown, which precluded the use of the native intact TA system in the study. No decrease in MICs was observed for strains carrying the cadmium-inducible TA system. This could be explained by the toxin-antitoxin ratio, which most likely remains the same as in the natural setup. Thus, over the short timescale (20 h) of the MIC assay, ectopic expression of the intact system does not result in a detectable decrease in MIC values. However, we recently showed that the growth of *S. aureus* expressing PemIK-Sa1 is significantly impaired at a subinhibitory antibiotic (chloramphenicol) concentration. As a consequence, such bacteria are rapidly outcompeted by those lacking the TA system when co-cultured (Bukowski et al., 2025). Moreover, the artificial activation of toxins from TA systems has been shown to be effective for the targeted killing of pathogens (Kang et al., 2020). Although our approach is experimentally suboptimal, it reveals the molecular mechanisms by which the TA system influences antibiotic resistance in these pathogenic bacteria.

In conclusion, we have shown that the expression of the PemK-Sa1 toxin reduces resistance to antibiotics from various chemical classes with different modes of action and molecular targets. This positions the toxin as a potential universal agent to combat multidrug-resistant bacteria, such as MRSA. Transcriptomics evidenced that PemK-Sa1 degrades not only gene transcripts encoding antibiotic resistance determinants but also alters those coding for proteins involved in processes vital to bacterial cells. This is reflected in the downregulation of a number of proteins, as evidenced by proteomics. Our findings suggest that artificial activation of TA systems might be a promising approach to reduce drug resistance and increase/restore the efficacy of antibiotics against bacterial pathogens.

## Data Availability

The datasets presented in this study can be found in online repositories. The names of the repository/repositories and accession number(s) can be found in the article/Supplementary material.
