# Supplementary material for: The toxin from the PemIK-Sa1 toxin-antitoxin system decreases Staphylococcus aureus resistance to antibiotics
Source: Front Microbiol. 2026 Jul 31;17:1863659. doi: 10.3389/fmicb.2026.1863659 (PMC13473009; doi:10.3389/fmicb.2026.1863659)
Supplement: Supplementary file 1 [file Data_sheet_1.zip › supplementary data/Supplementary data_v2.pdf]

Supplementary information for

The toxin from the PemIK-Sa1 toxin-antitoxin system decreases *Staphylococcus aureus* resistance to antibiotics

Emilia Bonar, Kinga Chlebicka, Michał Bukowski, Weronika Jodłowska, Maja Kosecka-Strojek, Anna Rabięcny, Piotr Suder, Małgorzata Hopciaś, Zuzanna Głowacka Grzyb, Viktoria Akkerboom, Andreas Voss, Artur J. Sabat, Benedykt Władyka

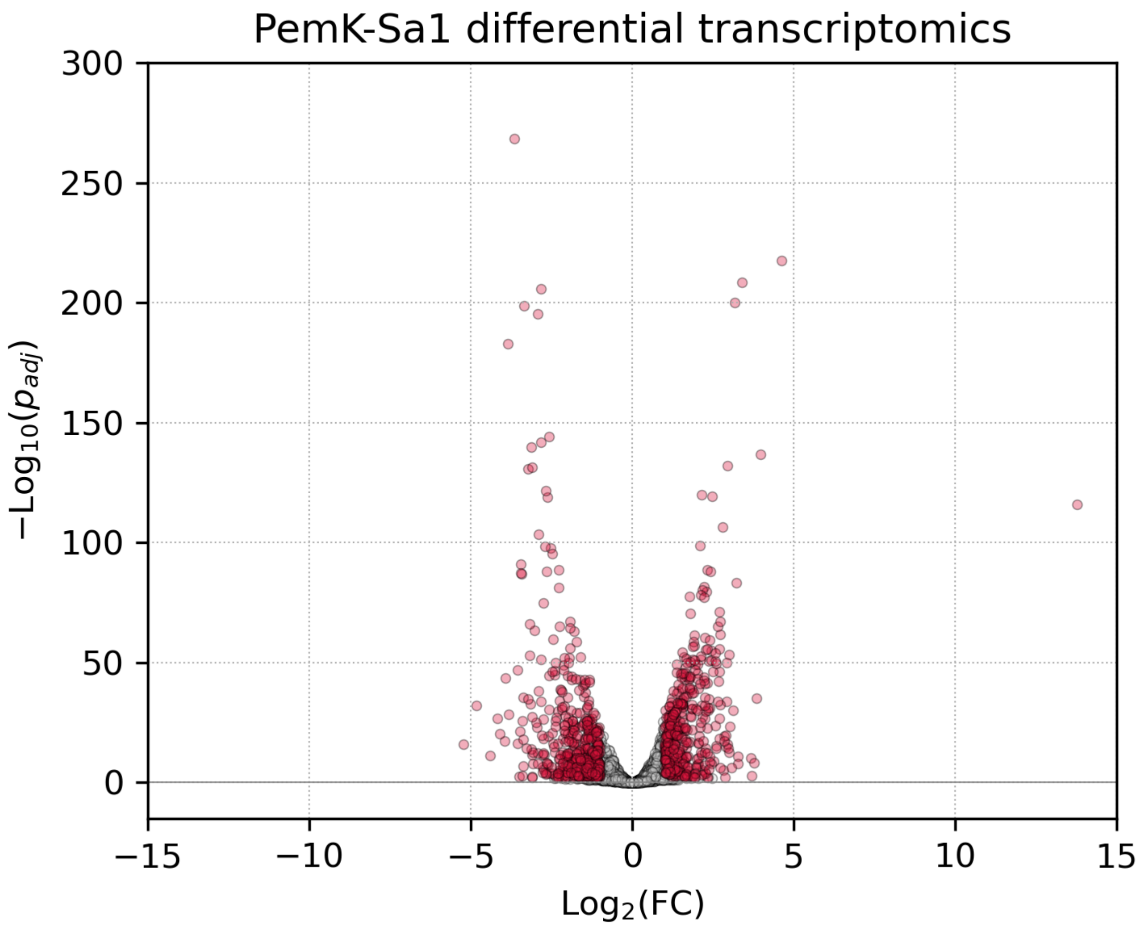

Supplementary figure S1. Volcano plot of differential gene expression in *S. aureus* 3956/13/K transformed with pCN51/pemKSa1. RNA for transcriptomic analysis was collected 45 minutes after inducing PemK-Sa1 toxin expression with cadmium ions. Non-significantly and significantly differentiating transcripts (fold change (FC) ≥2, p_{adj} <0.05) are marked with grey and red circles, respectively.

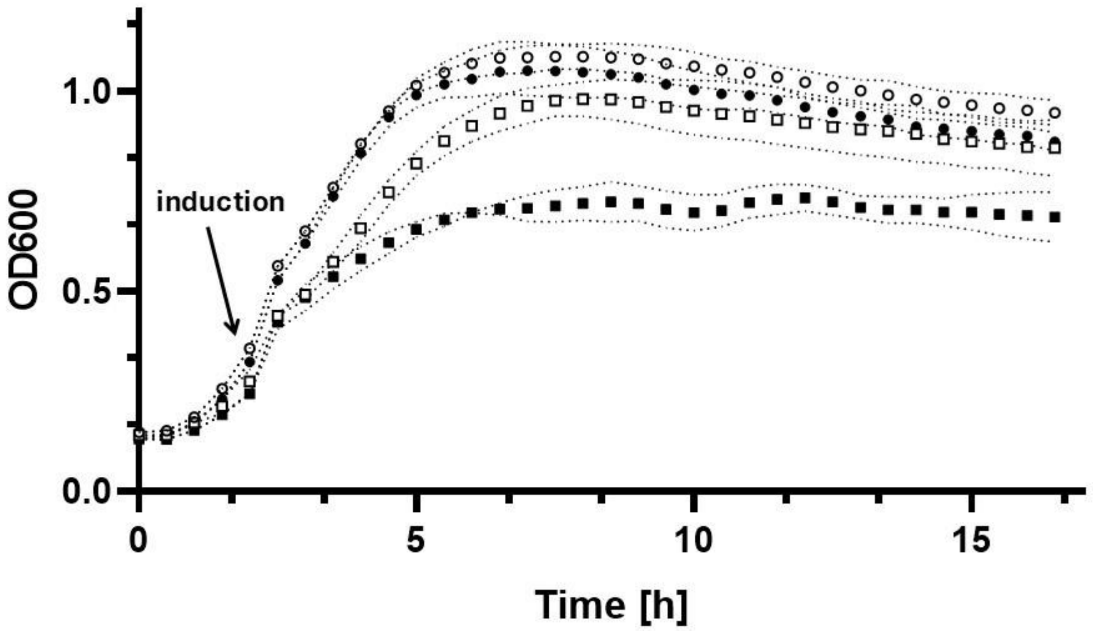

Supplementary figure S2. Growth curves of *S. aureus* 3956/13/K transformed with a plasmid carrying the *pemK-Sa1* toxin gene under a cadmium-inducible promoter (pCN51/pemKSa1) or a control (pCN51/Ø). The time of PemK-Sa1 expression induction is marked with an arrow. ○ 3956/13/K/pCN51/Ø, ● 3956/13/K/pCN51/Ø cadmium-induced, □ 3956/13/K/pCN51/pemKSa1, ■ 3956/13/K/pCN51/pemKSa1 cadmium-induced.

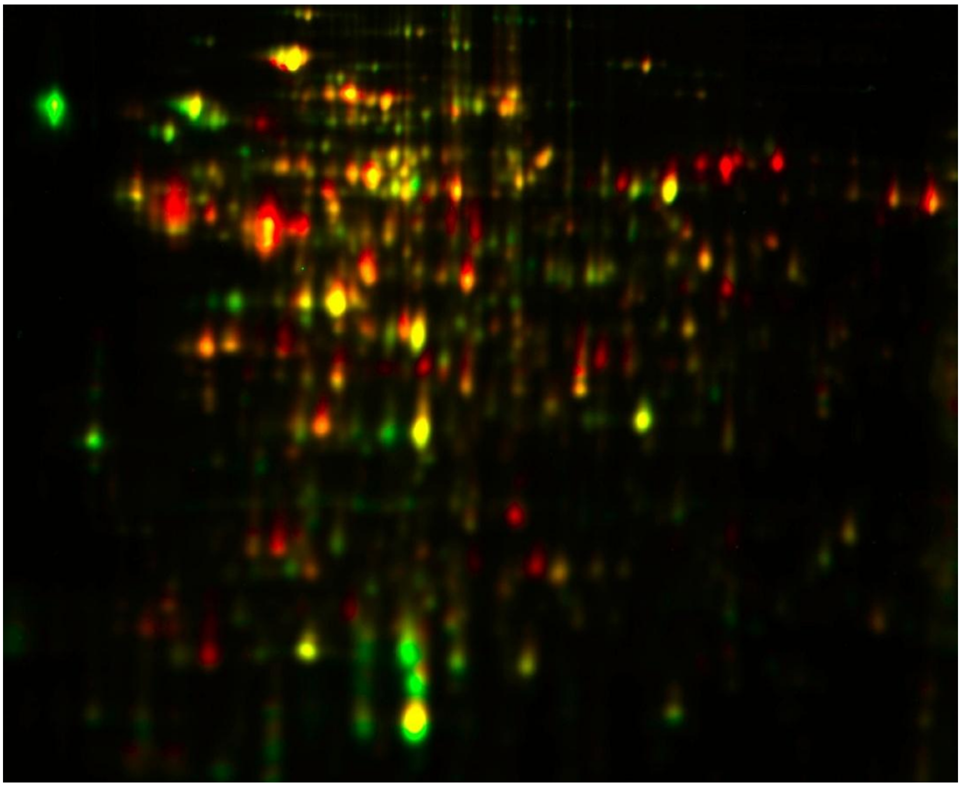

Supplementary figure S3. An example 2D-DIGE gel of cellular proteins from *S. aureus* 3956/13/K transformed with a plasmid carrying the *pemK-Sa1* toxin gene under a cadmium-inducible promoter (pCN51/pemKSa1) or a control (pCN51/Ø). Proteins were collected 90 minutes after toxin induction. The images were analyzed with DeCyder 7.2. Proteins that are positively and negatively differentiating in the PemK-Sa1 expressing strain are more intense in the green and red channels, respectively.

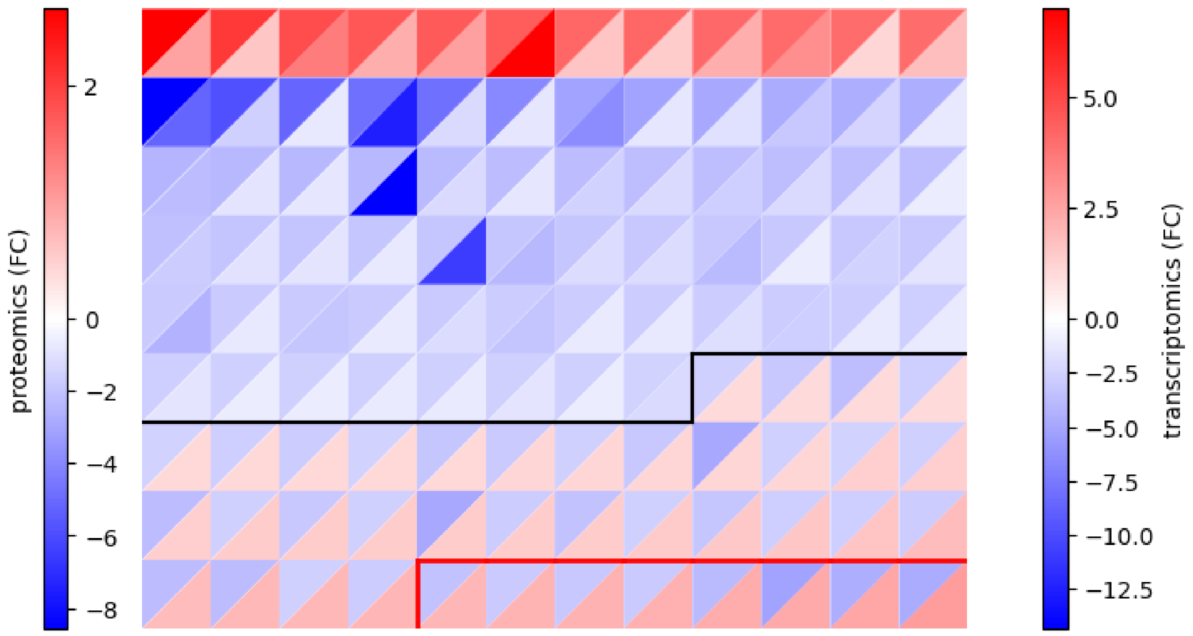

Supplementary figure S4. Heatmap illustrating the correlation between differential gene expression at the transcriptome and proteome levels. The upper and lower triangles in each square represent proteomics and transcriptomics data, respectively. Triangles of the same color within a square indicate the same direction of differential gene expression. The black curve separates genes that follow the same trend in both transcriptomics and proteomics. The red curve distinguishes genes that are strongly, inversely regulated between the two analytical techniques.

Supplementary table S1. MIC values for *S. aureus* strains transformed with a plasmid expressing intact PemIK-Sa1 TA system,PemK-Sa1 toxin, or carrying a control plasmid (Ø). Expression was induced by 0.5 µM $CdCl_2$.

Antibiotic	Strain	Biologi-cal replicate	MIC [µg/ml]	Mean	SD	Result
Penicillin	3956/13/K-Ø	I	900	1050	260	1050 ± 260
		II	900			
		III	1350			
	3956/13/K-pemK	I	55	52	10	52 ± 10
		II	40			
		III	60			
	3956/13/K-pemIK	I	1000	1067	58	1067 ± 58
		II	1100			
		III	1100			
Oxacillin	3956/13/K-Ø	I	264	292	24	292 ± 24
		II	306			
		III	306			
	3956/13/K-pemK	I	78	76	3	76 ± 3
		II	78			
		III	72			
	3956/13/K-pemIK	I	276	294	18	294 ± 18
		II	294			
		III	312			
Ciprofloxacin	3956/13/K pCN51/Ø	I	70	77	6	77 ± 6
		II	70			
		III	80			
	3956/13/K-pemK	I	20	13	6	13 ± 6
		II	10			
		III	10			
	3956/13/K-pemIK	I	130	130	0	130 ± 0
		II	130			
		III	130			
Chloramphenicol	3956/13/K-Ø	I	80	73	6	73 ± 6
		II	70			
		III	70			
	3956/13/K-pemK	I	40	33	6	33 ± 6
		II	30			
		III	30			
	3956/13/K-pemIK	I	70	70	0	70 ± 0
		II	70			
		III	70			
Kanamycin	3956/13/K-Ø	I	300	283	29	283 ± 29
		II	300			
		III	250			
	3956/13/K-pemK	I	100	117	29	117 ± 29
		II	150			
		III	100			

	3956/13/K-pemIK	I	400	333	58	333 ± 58
		II	300			
		III	300			
Fusidic acid	3956/13/K-Ø	I	40	43	5	43 ± 5
		II	50			
		III	40			
	3956/13/K-pemK	I	30	30	0	30 ± 0
		II	30			
		III	30			
	3956/13/K-pemIK	I	50	43	5	43 ± 5
		II	40			
		III	40			

Supplementary methods

Transcriptomic analysis, detailed description of total RNA isolation, mRNA purification, Next-Generation Sequencing, and gene expression analysis

4 ml of bacterial culture was centrifuged (5 min, 5000 g, 4 °C). RNA was isolated from the bacterial sediment using TrizolTM Reagent, Lysing Matrix B Tubes, and the Precellys Evolution homogenizer (Bertin Technologies) according to the following program: 4500 RPM speed for 10 cycles, 90 s each with 1 s break between cycles. Total RNA was treated with the On-Column DNase I Digestion Set (Sigma-Aldrich) and purified with the GeneJET RNA Purification Kit (Thermo Fisher Scientific). RNA quantity was measured with a Qubit 4.0 Fluorometer (Thermo Fisher Scientific), and quality was assessed using the RNA 6000 Nano LabChip Kit on a 2100 Bioanalyzer system (Agilent Technologies). Only samples with preserved 16S and 23S peaks and RIN values above 9 were selected for gene expression analysis. Total RNA was then enriched for bacterial mRNA using the RiboMinus Bacteria 2.0 Transcriptome Isolation Kit (Invitrogen) with 2.5 µg input RNA, and eluted in 25 µl of nuclease-free water. The rRNA depletion was confirmed with the RNA 6000 Nano LabChip Kit (Agilent Technologies). Libraries were prepared with the TruSeq Stranded mRNA kit (Illumina). The purified mRNA was quantified using a Qubit RNA HS assay kit and a Qubit 4.0 Fluorometer (Thermo Fisher Scientific), then diluted to 4 ng/µL. First and second strand cDNA synthesis was performed and purified using Agentcourt AMPure XP beads (Beckman Coulter Genomics). Subsequent adenylation of 3’ ends, ligation of multiple indexing adapters, and purification with AMPure XP beads were carried out. cDNA fragments were enriched via

PCR and purified. Fragment size distribution was checked with a 2100 Bioanalyzer using the Agilent High Sensitivity DNA Kit (Agilent Technologies), targeting 200-300 bp. Indexed libraries were normalized, pooled, and loaded onto an Illumina MiSeq reagent cartridge with the MiSeq reagent kit v3 (Illumina) for 150-cycle paired-end 2× 75 bp sequencing runs. Read quality was assessed with fastp 1.0 (Chen, 2025). Reads were then mapped to transcript sequences derived from S. aureus 3956/13K genome using Salmon 1.10 (Patro et al., 2017). Differential gene expression was modeled using Bioconductor DESeq2 1.42 (Love et al., 2014) and apeglm 1.24 (Zhu et al., 2019) libraries. The complete pipeline was executed using Nextflow 24.10 (DI Tommaso et al., 2017) and is available at https://github.com/michalbukowski/rnaseq-pipeline-2.

Proteomic analysis, detailed description of protein isolation, 2D-DIGE technique, and proteome analysis

Sample preparation and 2D DIGE proteomic analysis were performed as described by Bonar et al. (2020). For cellular protein isolation, 90 minutes after induction of PemK-Sa1 expression, bacteria were pelleted by centrifugation (5 min, 5000 g, 4 °C). The sedimented cells were washed twice with phosphate-buffered saline. Cells were suspended in Trizol™ Reagent and transferred to Lysing Matrix B Tubes according to the manufacturer’s instructions, and homogenized four times for 30 seconds at 6300 rpm using a Precellys Evolution homogenizer (Bertin Technologies). The supernatant was collected, proteins were isolated with TRI-Reagent, and solubilized in a lysis solution (7 M Urea, 2 M Thiourea, 30 mM Tris-HCl, 4% CHAPS). The protein concentration was determined using the Quick Start™ Bradford Protein Assay (Bio-Rad), and the appropriate amounts of protein from each sample were labeled with fluorescent tags using the G-Dyes Refraction-2DTM Labeling Kit (NH DyeAgnostics), which covalently bind to the ε-amino groups of lysines, enabling subsequent quantitative analysis. Samples were paired, mixed respectively, and protein separation was first conducted with isoelectrofocusing and then with SDS-PAGE according to Laemmli's (Laemmli, 1970) method. Gels were imaged with the ChemiDoc MP (Bio-Rad) device by exciting fluorescent tags with appropriate light wavelengths. The obtained proteome images were analyzed with DeCyder 7.2 software (GE Healthcare). To assess the significance of the results, the DeCyder program performed the Student's t-test and one-way ANOVA. Values with a significance level of ≤ 0.05 were considered statistically significant. The differentiating protein spots were excised from silver-stained gels (Shevchenko et al., 1996), hydrolyzed into peptides (Bonar et al., 2020), and

subjected to mass spectrometry identification. NanoLC-MS/MS analyses were performed as described by Lorencik et al. (2025) with minor modifications. Briefly, after destaining, protein reduction by DTT (5 mM, 30 min, 56°C), alkylation by iodoacetamide (10 mM, 30 min, RT, in darkness), proteolytic cleavage with trypsin (overnight, 37°C, mild shaking, Trypsin-Gold MS-grade, Promega, USA), samples were lyophilized and resuspended in 4% acetonitrile in water acidified with 0.1% formic acid (v/v). Peptides were then introduced onto the precolumn (300 µm ID, L=5 mm, Pepmap Neo Cartridge, Thermo Fisher Scientific, Lithuania), purified, and transferred to the capillary column (75 µm ID, 150 mm; both C18, 5 µm, Acclaim PepMap100, Thermo Fisher Scientific, Lithuania). The separation gradient was based on two solvents: water and acetonitrile, both acidified with 0.1% formic acid (v/v). The gradient parameters ranged from 5% to 30% acetonitrile over 33 minutes with flow rate 300 nl/min. All gradients were run using the Ultimate 3000 system (Thermo Fisher Scientific, Bremen, Germany) connected online to an Exploris 240 mass spectrometer (Thermo Fisher Scientific, Bremen, Germany). Basic settings of the mass spectrometer included: for MS1 scan range 350-1500 m/z; orbitrap resolution 60,000; RF Lens 67%; for MS2 (DDA approach) orbitrap resolution 60,000; scan range 200-2000; minimum ion intensity $8x10^5$; charge states 1-6; parent ion exclusion after 2 fragmentations; exclusion time 20 s; mass tolerance (high/low) 0.5 Da; centroided spectra. Raw data were converted into *.mgf files by RAW-converter (Scripps Institute, La Jolla, CA, USA) and searched using Mascot Search Engine (version 2.8.0) against two databases: Swiss-Prot and S_aureus_USA300. Settings of Mascot were as follows: enzyme: trypsin; up to 1 missed cleavage allowed; fixed modifications: carbamidomethylation; variable modifications: oxidation-Met; peptide tolerance: 50 ppm; #^{13}C=1; MS/MS tolerance: 50 ppm; instrument: ESI-FTICR. Final results with a score lower than 100 were omitted. If more than one protein with a score over 100 was identified in a single spot, further analysis was only conducted for proteins with a score up to 50% of the highest scoring protein. All proteomic data, including *.raw and *.mgf files and search results, are available in ProteomeXchange/PRIDE repository (Perez-Riverol et al., 2019) under accession number: PXD077076.

Supplementary references

Bonar, E., Chlebicka, K., Dubin, G., & Wladyka, B. (2020). Application of two-dimensional difference gel electrophoresis in identification of factors responsible for virulence of Staphylococcus aureus. In *Methods in Molecular Biology*. https://doi.org/10.1007/978-1-4939-9849-4_11

Chen, S. (2025). fastp 1.0: An ultra-fast all-round tool for FASTQ data quality control and preprocessing. *IMeta*, *4*(5). https://doi.org/10.1002/imt2.70078

DI Tommaso, P., Chatzou, M., Floden, E. W., Barja, P. P., Palumbo, E., & Notredame, C. (2017). Nextflow enables reproducible computational workflows. In *Nature Biotechnology* (Vol. 35, Number 4). https://doi.org/10.1038/nbt.3820

Laemmli, U. K. (1970). Cleavage of structural proteins during the assembly of the head of bacteriophage T4. *Nature*, *227*(5259), 680–685. https://doi.org/10.1038/227680a0

Lorencik, K., Ekiert, R., Pietras, R., Ner-Kluza, J., Hopciaś, M., & Osyczka, A. (2025). Defining the direct electron transfer connection between alternative complex III and cytochrome oxidase in *Flavobacterium johnsoniae*. *Biochimica et Biophysica Acta (BBA) - Bioenergetics, 1866*(2), 149548. https://doi.org/10.1016/j.bbabio.2025.149548

Love, M. I., Huber, W., & Anders, S. (2014). Moderated estimation of fold change and dispersion for RNA-seq data with DESeq2. *Genome Biology*, *15*(12). https://doi.org/10.1186/s13059-014-0550-8

Patro, R., Duggal, G., Love, M. I., Irizarry, R. A., & Kingsford, C. (2017). Salmon provides fast and bias-aware quantification of transcript expression. *Nature Methods*, *14*(4). https://doi.org/10.1038/nmeth.4197

Perez-Riverol, Y., Csordas, A., Bai, J., Bernal-Llinares, M., Hewapathirana, S., Kundu, D. J., Inuganti, A., Griss, J., Mayer, G., Eisenacher, M., Pérez, E., Uszkoreit, J., Pfeuffer, J., Sachsenberg, T., Yilmaz, Ş., Tiwary, S., Cox, J., Audain, E., Walzer, M., … Vizcaíno, J. A. (2019). The PRIDE database and related tools and resources in 2019: Improving support for quantification data. *Nucleic Acids Research*, *47*(D1), D442–D450. https://doi.org/10.1093/nar/gky1106

Shevchenko, A., Wilm, M., Vorm, O., & Mann, M. (1996). Mass spectrometric sequencing of proteins from silver-stained polyacrylamide gels. *Analytical Chemistry*, *68*(5), 850–858. https://doi.org/10.1021/ac950914h

Zhu, A., Ibrahim, J. G., & Love, M. I. (2019). Heavy-Tailed prior distributions for sequence count data: Removing the noise and preserving large differences. *Bioinformatics*, *35*(12). https://doi.org/10.1093/bioinformatics/bty895
